# The effects of exercise therapy feedback on subjective treatment outcome and patient satisfaction: study protocol for a mono-centric, randomized, controlled trial in orthopedic rehabilitation (FeedYou)

**DOI:** 10.1186/s13102-023-00626-2

**Published:** 2023-02-08

**Authors:** André Arik Schuber, Sebastian Schmidt, Sarah Hombach, Andrea Schaller

**Affiliations:** 1 Working Group Physical Activity-Related Prevention Research, Institute of Movement Therapy and Movement-Oriented Prevention and Rehabilitation, German Sport University Cologne, Am Sportpark Müngersdorf 6, NawiMedi, Ground Floor, 50933 Cologne, Germany; 2 Therapy Department, Aggertalklinik, Engelskirchen, Germany

**Keywords:** Exercise therapy, Feedback, Subjective treatment outcome, Patient satisfaction, Study protocol, Randomized controlled trial

## Abstract

**Background:**

The disease burden of musculoskeletal disorders necessitates multidisciplinary and patient-centered models of care. Exercise therapy represents a first-line treatment strategy and a central component of medical rehabilitation. In order to realize the goals of long-term physical activity and participation as proposed by the ICF, exercise therapy can be supplemented by interventional techniques from the field of psychotherapy. Although psychotherapist feedback has been shown to improve therapeutic outcome and patient satisfaction, feedback use in exercise therapy is mostly limited to motor learning and exercise instruction. The present paper therefore describes the use of multidimensional exercise therapy feedback in medical rehabilitation. The aims of the trial presented in this study protocol are to evaluate the effects of this novel feedback approach on rehabilitation outcomes in comparison to usual care.

**Methods:**

The study is designed as a prospective, mono-centric, randomized controlled, superiority trial (RCT) with two parallel groups and three measuring points: T0 = start of three-week inpatient rehabilitation, T1 = end of three-week inpatient rehabilitation, T2 = 12-week follow-up. In total, 132 patients suffering from chronic neck, shoulder and/or lumbar spine disorders will be recruited. The intervention involves multidimensional exercise therapy feedbacks during the initial and final physical therapist examination, as well as short exercise therapy feedbacks during the course units of the mandatory group-based exercise therapy program. Primary outcomes are the subjective treatment outcome, assessed by BPI and indication-specific questionnaires, as well as patient satisfaction, assessed by ZUF-8 and an intervention-specific questionnaire. The final data collection is expected by May 2023.

**Discussion:**

This study may provide a valuable insight into the effectiveness of multidimensional exercise therapy feedback to improve treatment outcomes and patient satisfaction in medical rehabilitation. This could contribute to rehabilitation quality assurance and the long-term physical activity behavior of rehabilitation patients.

*Trial registration* The trial has been registered with the German Clinical Trial Register (DRKS) under the Registration Number DRKS00027263.

## Background

Musculoskeletal disorders including low back pain and osteoarthritis represent a large disease burden and in 2017 affected approximately 1.3 billion individuals globally [1]. Often resulting in functional decline, a loss of social and occupational participation and a reduced well-being [2], these disorders are further exacerbated by aging, obesity and sedentary lifestyles [3, 4]. In addition to the personal impact, the economic consequences of productivity loss and medical expenses are very high [5] and should be addressed by multidisciplinary, patient-centered models of care [2].

In Germany, medical rehabilitation programs represent an important sector of the health care system and are mainly provided by the German pension insurance and the statutory health insurance [6]. Following a multidisciplinary approach, these programs incorporate a range of biomedical, psychosocial and educational services with a clearly defined frequency and duration [7]. In line with the model of patient-centered-care [8] and the International Classification of Functioning, Disability and Health (ICF), medical rehabilitation in Germany aims to improve the patient’s functional capacity with the goal of enabling activities and increasing participation [9]. Accordingly, the integration of the patient’s view in the evaluation of rehabilitation outcomes constitutes a key quality criterion and is operationalized via patient-reported outcomes including patient satisfaction and subjective treatment outcome [10].

Since regular physical activity and exercise have been shown to improve functional capacity and reduce disability in several musculoskeletal disorders [11], exercise therapy (ET) represents a cornerstone of many European medical rehabilitation programs [12]. For example, ET accounts for more than a third (35%) of therapeutic services provided in German medical rehabilitation settings [13, 14]. Covering physical training as well as psychosocial and educational goals [15, 16], ET in Germany is usually planned and carried out by trained sports scientists and physical therapists and organized as either an individual or group-based treatment [17]. Furthermore, since ET contains the most extensive patient-therapist interaction in German medical rehabilitation [13], a corresponding impact of ET on patient-reported outcomes can be assumed. However, despite extensive data on the biomedical effects of ET [18], little is known about the influence of the therapist’s behavior and the patient-therapist interaction on patient satisfaction and subjective treatment outcome in medical rehabilitation.

Research from the field of psychotherapy has highlighted therapist feedback as a versatile and powerful interventional technique [19]. In this context, feedback can be understood as information provided by an agent (e.g. therapist) regarding aspects of a person’s performance, behavior and/or understanding [20, 21]. In particular, regular psychotherapist feedback on the patient’s progress has been associated with an improved therapeutic outcome, better patient-therapist interaction, and higher levels of patient satisfaction [22–24]. In contrast, research on feedback use in ET is mostly limited to motor learning and exercise instruction [25–27], disregarding important psychosocial dimensions like motivation, self-regulation and volition. To our knowledge, no studies have investigated the effects of a multidimensional ET feedback incorporating biomedical and psychosocial content. Therefore, the present article describes the study protocol for a randomized controlled trial to evaluate the effects and the patients’ acceptance of multidimensional ET feedback in medical rehabilitation. In this context, the following primary research question will be addressed:

- How does multidimensional exercise therapy feedback affect the subjective treatment outcome and satisfaction of rehabilitation patients with chronic disorders of the neck, shoulder and/or lumbar spine?

Related to the primary research question, the following hypotheses were developed:

1. In the intervention group, the general subjective treatment outcome measured with the Brief Pain Inventory is on average 2.0 points below the control group at the end of the inpatient rehabilitation program (resp. at 12-week follow-up).
2. The intervention group shows better indication-specific subjective treatment outcomes than the control group at the end of the inpatient rehabilitation program (resp. at 12-week follow-up).
3. The intervention group shows higher levels of satisfaction with rehabilitation than the control group at the end of the inpatient rehabilitation program (resp. at 12-week follow-up).

Additionally, the following secondary research questions concerning the project’s process quality are evaluated by an explanatory approach:

- How do rehabilitation patients and exercise therapists rate the acceptance and feasibility of multidimensional exercise therapy feedback?
- What factors influence the patient’s work ability at the end of the inpatient rehabilitation program (resp. at 12-week follow-up)?
- What are subjective outcome and satisfaction criteria of rehabilitation patients?

## Methods

### Study design

The FeedYou study is designed as a prospective, mono-centric, randomized controlled, superiority trial (RCT) with two parallel groups and three measuring points: T0 = start of three-week inpatient rehabilitation, T1 = end of three-week inpatient rehabilitation, T2 = 12-week follow-up (Table 1). The study is registered in the German Clinical Trial Register (DRKS00027263) and approved by the German Sport University Research Ethics Committee (#179/2021, approval date February 10, 2022). It has undergone external peer-review by the funding body refonet—Rehabilitation research network of Deutsche Rentenversicherung Rheinland (German Pension Insurance). The recruitment of patients started in July 2022 and the T1 measurement is planned to finish in February 2023. The 12-week follow-up (T2) will be completed in May 2023. This study protocol is designed according to the SPIRIT 2013 Statement: Defining Standard Protocol Items for Clinical Trials [28].

**Table 1:**
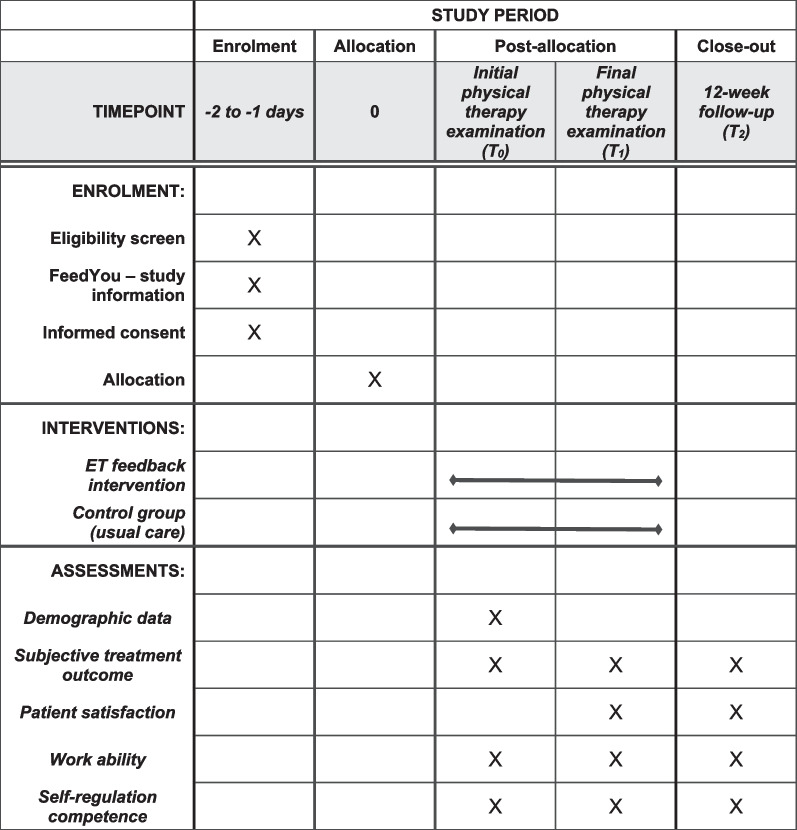
SPIRIT timetable

### Setting and participants

Participants are recruited from an inpatient rehabilitation clinic in North Rhine-Westphalia, Germany. To be eligible to participate in this study, patients are required to be at least 18 years old and to meet the following criteria: (1) suffering from neck, shoulder and/or lumbar spine disorders over a period of more than three months, (2) scheduled participation in the inpatient orthopedic rehabilitation program and (3) sufficient knowledge of the German language. Exclusion criteria are: (1) acute herniated discs, (2) postoperative conditions following neck, shoulder and/or lumbar spine surgery less than two months ago, or (3) language barriers. The eligibility criteria for the therapists performing the intervention are: (1) an ET-related qualification (e.g. certified sports scientist or physical therapist) and (2) a minimum of three-year experience with group ET treatments (e.g. back schools, aquatic therapy or medical nordic walking). The patients are informed by the clinic staff about the study’s content, design and eligibility criteria during the welcoming event at the start of the rehabilitation program. Individuals who are interested in participating are required to provide written informed consent. The participants receive no financial compensation for their participation in the study. The coordinating investigator (AAS) randomly assigns the participants to the intervention group or the control group following a 1:1 allocation using blocked randomization. The corresponding block size is independently provided by the funding body refonet and is not disclosed to ensure concealment. The coordinating investigator (AAS) holds the randomization key. All data related to personal information are pseudo-anonymized. Although participants are blinded to the allocation, blinding of the clinic staff is impossible due to the content of the intervention.

### Intervention

The intervention consists of multidimensional ET feedbacks during the initial and final physical therapist examination, as well as short group ET feedbacks during the course units of the mandatory group ET program (shoulder-back school). The respective feedbacks are based on the feedback model of Hattie & Timperley [20], whose four levels (*task, process, self-regulation* and *self*) were adapted to the contents and goals of ET during medical rehabilitation (Table 2). It was developed together with experts from health psychology and medical rehabilitation and was implemented as a structured and partially standardized ET feedback curriculum.

**Table 2:** Description of ET feedback levels (adapted from Hattie & Timperley [20])

Level of feedback	Definition	Adaptation to ET
Task level	Feedback about how well a task is being accomplished or performed	Feedback about the patient’s physical performance and level of disability and discomfort
Process level	Feedback about the processes underlying a task and corresponding error correction strategies	Feedback about the patient’s exercise execution and the ability to recognize and respond to errors in exercise execution
Self-regulation level	Feedback about the way a person monitors, directs, and regulates their actions toward the learning goal	Feedback about the patient’s ability to monitor, control and regulate their own training and learning processes
Self level	Personal evaluation by the agent (e.g. teacher, therapist)	Personal evaluation related to the patient’s self-regulation, willingness to exert effort and/or commitment during exercise therapy

The *first multidimensional ET feedback* is provided during the initial 30-min physical therapy examination at the start of the inpatient rehabilitation program. Following an initial interview to determine the patient’s physical activity level and preferences, the patient’s current physical condition is determined through objective assessments (handgrip strength, joint position sense and postural control) as well as patient-reported outcome measures (PROMs) on indication-specific pain and disability (Table 3). Based on these data, the exercise therapist provides the patient with a detailed, visual and verbal feedback pertaining to the task level (Table 2) and helps to determine ET-specific rehabilitation goals.

**Table 3:** Instruments of quantitative evaluation

	Instrument	Time of measurement
*Primary outcome measures*		
Subjective treatment outcome (generic)	BPI [29, 30]	T0, T1, T2
Subjective treatment outcome (indication-specific)	NPAD [33, 34]	T0, T1, T2
	QuickDASH [35]	T0, T1, T2
	RMDQ [36, 37]	T0, T1, T2
Patient satisfaction	ZUF-8 [38]	T1, T2
	Unstandardized, intervention-specific questionnaire	T1, T2
*Secondary outcome measure*		
Self-regulation competence	Physical activity-related health competence questionnaire (Sub-dimension: self-regulation competence) [42, 43]	T0, T1, T2
*Person-related variables*		
Age, sex, education level, employment situation	Demographic standards [44]	T0
Job profile	GEDA 2019/2020-EHIS [45, 46]	T0
*Process quality measures*		
Acceptance and feasibility of ET feedback	Unstandardized, intervention-specific questionnaire	T1
Work ability	WAS [40, 41]	T0, T1, T2

T0 = Start of inpatient rehabilitation; T1 = End of inpatient rehabilitation; T2 = 12-week follow-up

The short *group ET feedbacks* are provided by the therapist at the end of every shoulder-back school course unit. The shoulder-back school combines educational and exercise components, with the goal of informing patients about anatomy and physiology as well as offering advice on how to prevent and manage pain [9]. It consists of ten course units on consecutive days, each lasting between 30 to 45 min. Every group ET feedback consists of a three-minute evaluation regarding the group’s rehabilitation progress exemplified by the performance, participation and commitment of individual group members.

The *second multidimensional ET feedback* is provided during the final 30-min physical therapy examination at the end of the inpatient rehabilitation program. After an initial interview to determine the patient’s general opinion regarding the program, the assessments and PROMs from the initial examination are repeated to determine the objective and subjective treatment effects. Employing all four levels of ET feedback (Table 2), the therapist then provides the patient with a visual and verbal summary of their performance and individual progress during the inpatient rehabilitation program. Additionally, possibilities for the continuation of training are discussed to facilitate long-term physical activity and social participation following the program.

The control group (usual care) receives two physical therapist examinations lasting 30 min each and including PROMs on pain and disability. In contrast to the intervention group, no objective assessments are performed and the therapist only gives a generic feedback on the patient’s current condition, rehabilitation goals and progress. The control group also receives the shoulder-back school, which is matched in content and duration to the intervention group, but does not include group ET feedbacks.

### Quantitative data collection

Data related to the primary research question are collected by PROMs at each of the three measurement points (Table 1). The T0 and T1 measurements take place during the respective initial and final physical therapy examinations at the start and end of the three-week inpatient rehabilitation program. The questionnaires for the 12-week follow-up (T2) are sent to the patient’s home, and a reminder is sent if the questionnaires are not returned within two weeks.

In order to test hypothesis 1, the generic subjective treatment outcome is operationalized using the Brief Pain Inventory (BPI) [29, 30]. The BPI measures pain severity as well as pain interference with daily activities. Its rating scales have high construct validity (Factor analysis results: 0.5–0.91) [31] and reasonable internal consistency (Cronbach’s alpha = 0.84) [32]. Furthermore, the BPI shows adequate test–retest reliability (Pearson correlation r = 0.97) [30] and is sensitive to change [31].

For the evaluation of hypothesis 2, indication-specific subjective treatment outcomes are operationalized with the Neck-Pain and Disability Scale (NPAD) [33, 34], the shortened Disabilities of the Arm, Shoulder and Hand Questionnaire (QuickDASH) [35] as well as the Roland and Morris Disability Questionnaire (RMDQ) [36, 37].

Patient satisfaction (hypothesis 3) is evaluated using the ZUF-8 [38], a German version of the Client Satisfaction Questionnaire (CSQ-8) [39] adapted for rehabilitation. In addition, an unstandardized intervention-specific questionnaire is used to assess the patient’s satisfaction with the physical therapy examinations and group ET program (Table 3).

The secondary research questions relating to the project’s process quality are investigated using an unstandardized, intervention-specific questionnaire to evaluate the acceptance and feasibility of multidimensional ET feedback from the patients and therapists’ view. In addition the Work ability score (WAS) [40, 41] is employed to allow for the identification of work-influencing factors of rehabilitation patients using multi-level regression analysis.

### Sample size calculation

The power calculation was based on hypothesis 1. Due to insufficient evidence for the effectiveness of the multidimensional ET feedback, the sample size was calculated based on a minimal clinically important difference of 2.0 points (SD = 2.5 points) between groups in the primary outcome as measured by the BPI [47]. Given a power of 0.9 with a one-sided test and an alpha of 0.05, the calculated total sample size was 66 participants (33 per group). Anticipating a maximum loss to follow-up of about 50%, the calculated target sample size was 132 patients.

### Statistical analysis

Descriptive statistics will be used to describe the main characteristics of the study population. For the primary research question, data will be analyzed according to the complete case principle. In addition, an intention-to-treat analysis will be performed to explore the robustness of the results. Group differences will be tested using multi-level regression analysis taking into account baseline data on age and gender. The significance level will be adjusted for multiple testing. A dropout analysis will be conducted to control for differential attrition bias. All data analysis will be carried out using SPSS (Version 29, IBM, Armonk, NY, USA).

### Qualitative data collection

The qualitative evaluation comprises semi-structured interviews with patients at the end of the inpatient rehabilitation program (T1). Objectives of the qualitative evaluation are the investigation of the therapists and physicians’ impact on treatment outcome and patient satisfaction. In addition, rehabilitation success criteria will be examined from the patients’ point of view. Key questions to determine these patient-centered quality criteria will be derived from previous quantitative and qualitative assessments of patients’ opinions and needs in musculoskeletal rehabilitation [48–50]. By the use of open-ended questions, we expect to gain a more detailed insight into the value of patient-therapist interaction and effects of therapist feedback on patient satisfaction. Prior to data collection, the wording and relevance of the interview questions will be reviewed by an experienced researcher (AS). In addition, the interview guide will be tested using two pilot interviews with rehabilitation patients from the intervention group to ensure comprehensibility and determine the interview duration. The interviews will be conducted in German and lead by one researcher (AAS) to ensure consistency of questioning. Although the exact sample size will be based on theoretical saturation, we plan to include a minimum of 10 to 12 patients. The interviews will be audio-recorded and transcribed verbatim by a professional typist using the system of Dresing & Pehl [51]. The transcribed data will then be analyzed using structuring content analysis [52] with the software MAXQDA (Version 2020, VERBI GmbH, Berlin, Germany).

## Discussion

Our study evaluates the implementation and effects of a novel, multidimensional ET feedback and contributes to quality assurance in medical rehabilitation with regard to the process and outcome quality dimensions.

Several operational challenges have to be taken into account during the execution of this study. First, the COVID-19 pandemic might influence the recruitment of study participants. Although the length of the recruitment period was based on the number of eligible patients taking part in the clinic’s rehabilitation program in 2019, 2020 and 2021, COVID-19 might impede patient participation and lead to unexpected dropouts. Therefore, the recruitment period might have to be extended in order to achieve the target sample size. Secondly, the content of the intervention might be influenced by the personal characteristics and social-communicative abilities of the respective exercise therapist performing the ET feedback. Therefore, therapists are required to adhere to the structure and content of the partially standardized ET feedback curriculum. In addition, only two exercise therapists are responsible for the intervention and control group, respectively, thereby minimizing ET feedback variability.

There are several strengths in the design of this study. We expect that the combination of quantitative and qualitative data collection will not only identify the potential effects of multidimensional ET feedback (outcome quality), but also highlight possible difficulties and shortcomings during the implementation of the intervention (process quality). Furthermore, the 12-week follow-up will provide data on the medium-term effectiveness of ET feedback following medical rehabilitation. Finally, the wide spectrum of instruments measuring treatment outcome, patient satisfaction, self-regulation and work ability will allow for the examination of factors influencing participation as described by the ICF model.

Since the clinic’s exercise therapists are responsible for delivering the intervention, this study is limited to a single-blind design. Although both the intervention and control group are organized as closed groups, participant blinding might theoretically be compromised through communication between groups during mealtime or recreational activities. Furthermore, the study-specific information provided to patients during the welcoming event might cause a selection bias and necessitate additional participant recruitment. Finally, due to clinic-specific operational procedures, patient randomization and group allocation has to be performed several days prior to participant recruitment during the welcoming event.

Based on the results of the planned study, multidimensional ET feedback implementations for further indications and rehabilitation clinics might be developed.

## Data Availability

Not applicable.

## Abbreviations

BPI: Brief Pain Inventory
CSQ-8: Client Satisfaction Questionnaire
DRKS: German Clinical Trial Register
ET: Exercise therapy
ICF: International Classification of Functioning, Disability and Health
NPAD: Neck-Pain and Disability Scale
PROMs: Patient-reported outcome measures
QuickDASH: Shortened Disabilities of the Arm, Shoulder and Hand Questionnaire
RCT: Randomized controlled trial
RMDQ: Roland and Morris Disability Questionnaire
SD: Standard deviation
SPIRIT: Standard Protocol Items—Recommendations for Interventional Trials
WAS: Work Ability Score
ZUF-8: Questionnaire to Evaluate Patient Satisfaction
